# Supplementary material for: Unipolar quantum optoelectronics for high speed direct modulation and transmission in 8–14 µm atmospheric window
Source: Nat Commun. 2024 Sep 13;15:8040. doi: 10.1038/s41467-024-52053-7 (PMC11399236; doi:10.1038/s41467-024-52053-7)
Supplement: Supplementary file 1 — Supplementary Information [file 41467_2024_52053_MOESM1_ESM.pdf]

Unipolar Quantum Optoelectronics for High Speed Direct Modulation and Transmission in 8-14 µm Atmospheric Window

Hamza Dely[1,8]*, Mahdieh Joharifar[2,8], Laureline Durupt[3,8], Armands Ostrovskis[4], Richard Schatz[2], Thomas Bonazzi[1], Gregory Maisons[3], Djamal Gacemi[1], Toms Salgals[4], Lu Zhang[5], Sandis Spolitis[4], Yan-Ting Sun[2], Vjačeslavs Bobrovs[4], Xianbin Yu[5], Isabelle Sagnes[6], Konstantinos Pantzas[6], Angela Vasanelli[1], Oskars Ozolins[7,4,2], Xiaodan Pang[2,4,7]* and Carlo Sirtori[1]*

[1]Laboratoire de Physique de l'ENS, Département de Physique, École Normale Supérieure, Université PSL, Sorbonne Université, Université Paris Cité, CNRS, 75005 Paris, France. [2]Department of Applied Physics, KTH Royal Institute of Technology, 106 91 Stockholm, Sweden. [3]mirSense, 2 Bd Thomas Gobert 91120 Palaiseau, France. [4]Institute of Telecommunications, Riga Technical University, 1048 Riga, Latvia. [5]College of Information Science and Electrical Engineering, Zhejiang University, Hangzhou 310027, China. [6]RISE Research Institutes of Sweden, 164 40 Kista, Sweden. [7]These authors contributed equally: Hamza Dely, Mahdieh Joharifar, Laureline Durupt. *Corresponding Authors, email: hamza.dely@ens.fr, xiaodan@kth.se, carlo.sirtori@ens.fr.

Supplementary Information

1) The generalized operational principle of QCL, QWIP and QCD

Quantum cascade laser (QCL) based on inter-subband transition was firstly demonstrated in 1994[1], following the previous ideas of superlattices[2] and light amplification in heterostructure[3]. An illustration of a typical QCL heterostructure design under bias current is shown in Supplementary Fig. 1. The optical transition occurs in the central part of the structure, where is surrounded by digitally graded alloys that form minibands. These minibands are engineered to guide electrons in the same direction as the electric field under a bias, moving from the top to the bottom of the miniband. The left miniband is known as the injector, which is depicted as the left grey band in the figure. Close to the end of the injector edge, electrons are transferred to the top energy level of the radiative transition. Following the emission of a photon, which demotes the

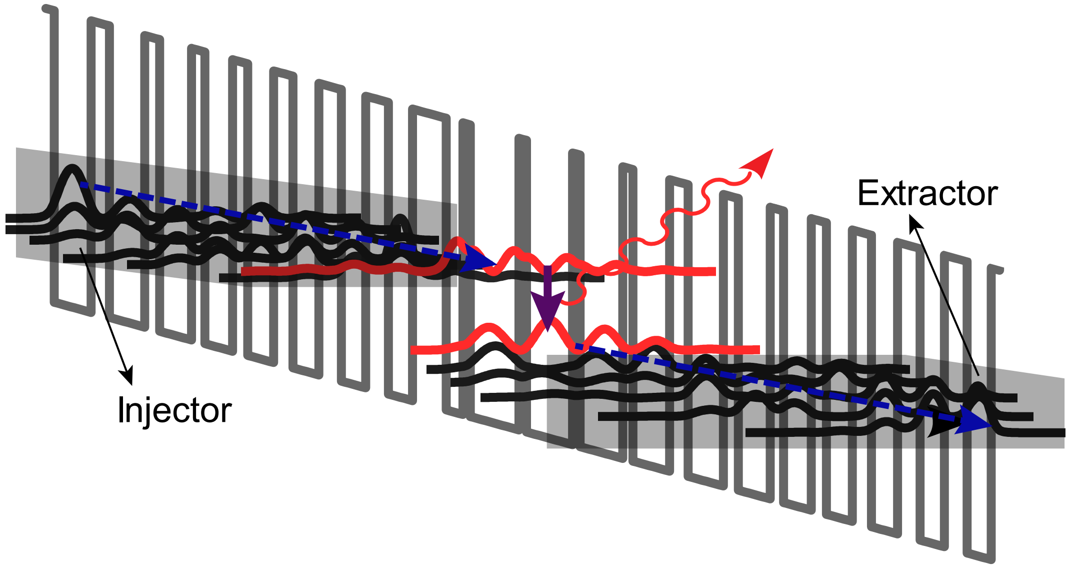

Supplementary Fig. 1. Illustration of a QCL heterostructure design subjected to bias. The left miniband (depicted as the left grey band containing black states), termed the injector, channels the electron via non-radiative processes and resonant tunnelling to the upper level of the radiative transition (highlighted in red). Following this, the electron transitions to the lower state, emitting a photon in the process, and then tunnels into the right miniband, designated as the extractor. Given the repeated nature of the structure, the extractor concurrently serves as an injector for the subsequent radiative transition.

electron to a lower energy level, a phonon-assisted transition occurs, transferring the electron to the next miniband, termed the extractor. This structural pattern is recurrent, with the extractor also functioning as the injector for the subsequent period, effectively recycling the electron. This mechanism significantly enhances the quantum efficiency and the overall gain of the heterostructure. It's worth noting that all electron movements within the structure are facilitated by tunnelling from one well to another through the barriers. In summary, such a heterostructure design facilitates inter-subband transitions, covering wide wavelength range from the mid-IR to the THz regime, potentially supporting wavelength division multiplexing (WDM). Moreover, it has intrinsic merit for broadband modulation due to the low carrier lifetime, thus the absence of relaxation oscillation-induced first-order frequency roll-off.

Quantum well infrared photodetector (QWIP) was one of the first unipolar quantum optoelectronic devices developed for mid-infrared applications. The first reported QWIP based on GaAs/AlGaAs was in 1987 by Levine *et al.*[4]. A typical single-period QWIP structure without and with bias applied are depicted in Supplementary Fig. 2a and b, respectively. As one can see from the figure, in this device, the GaAs well contains two engergy levels, i.e., a low-energy level that is well-confined, and a second energy level located close to the edge of the AlGaAs barrier. When an incoming infrared photon excites the electron from the lower level to the upper level, there is a probability that the excited electron can escape to the contrinuum due to thermal excitation and scattering. However, if there is no bias applied to the structure, the electron moves isotropically with zero mean current. Therefore, to generate photocurrent, a bias is necessary, as shown in Supplementary Fig. 2b. In this way, the isotropy is converted to directional electron displacement, creating a net non-zero current. In addition to thermal excitation, the barrier on one side of the well is reduced, permitting electrons to tunnel directly from the upper energy level to the continuum.

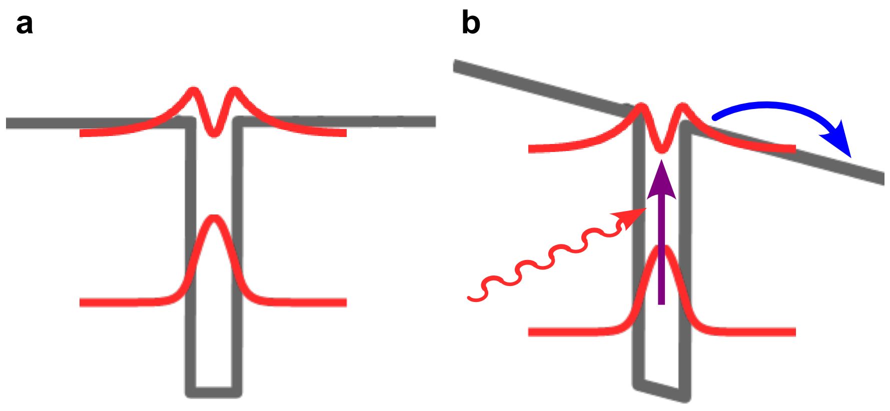

Supplementary Fig. 2. Illustration of a single period QWIP. a. No bias applied. b. Bias voltage applied.

With a cascading design where such single well unit is repeated multiple times, the number of carriers available for transport can increase substantially to improve on the photocurrent. In this way, the detector could reach a high responsivity level when operating at low temperatures (below 80 K). However, its performance drops drastically at higher temperatures, and may decrease by orders of magnitude in terms of responsivity at room temperature. Recently, a novel approach has been proposed to enhance the performance of QWIP by inserting the heterostructures into patch antenna resonator arrays, forming a metamaterial structure[5]. Two major benefits from such a design are: 1) the heterostructure is exposed to a significantly enhanced incoming mid-infrared electric field, resulting an increase in the photocurrent, a phenomenon referred to as the antenna effect; and 2) the electrical surface is reduced, which limit the current noise generated by the device and the capacitance is simultaneously reduced. Therefore, this design improves on both signal-to-noise ratio (SNR) and bandwidth of the detector, equipping it with high-speed potential. In our work, we adopted metamaterial-assisted stripe design to achieve high responsivity and high bandwidth, enabling us achieving high-speed free-space data transmission in the mid-infrared.

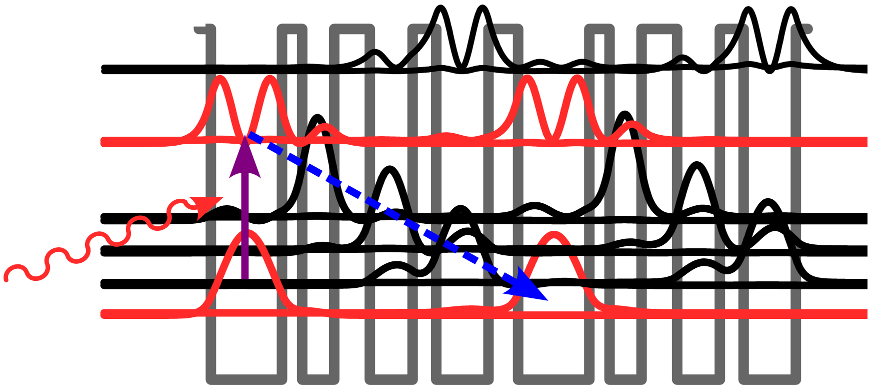

Supplementary Fig. 3. Illustration of a vertical QCD architecture. The primary well showcases two levels (highlighted in red) that dictate the device's absorption energy. When an electron occupies the excited state, it has the potential to tunnel through the barrier into the subsequent well, thereafter transitioning through the series of black-state levels to the ensuing period. Notably, this entire mechanism operates without the need for an electrical bias.

The concept of quantum cascade detector (QCD) was firstly explored in the early 2000's by directly using a QCL as a detector[6]. Then it was proposed as a new type of QWIP based on electronic tunnelling through the barriers similar to QCL[7]. A generic QCD architecture consisting of two periods is sketched in Supplementary Fig. 3. Each period consists of two major parts, i.e., a broad well and a sequence of narrow wells. The first broad well locates at the beginning of the structure with two states highly localized. These states are involved in the vertical radiative transition that defines the device's absorption peak. Following this well, a sequence of narrow wells with progressively increasing size are placed. Such a configuration is designed to create a ladder used by the electrons to relax non-radiatively through resonant tunnelling to the next period, instead of relaxing to the ground state within the same period. The extraction into the next period becomes more efficient as the energy states of the ladder come closer together. An improvement of the design was proposed in 2014 by using the first two wells, instead of the first well only, to create a diagonal radiative transition[8]. In this way, the electron can be extracted to the ladder more

efficiently, thus enhancing the responsivity of the detector. Such as design is also more robust to fabrication defects as it relaxes the strict alignment requirement of the resonant tunnelling.

The main features of the QCD include small footprint, zero-bias, and room-temperature operation. Compared with the QWIP where the structure asymmetry is induced by the biasing electrical field, the QCD has intrinsic asymmetry in its structure design. In this way, the photoexcited electrons flow naturally following the orientation and generate a non-zero net photocurrent. Further, as the QCDs rely on resonant tunnelling for electron transport, they have almost zero dark current and can provide high SNR. This property enables their room-temperature operation. However, high fabrication precision of QCD considering its complex multi-layer designs is typically challenging, its responsivity is normally limited in practice. Similar with the recent progress in QWIP, a novel approach to improve the QCD responsivity is to embed the detector in antenna to form a metamaterial-patterned device[9], which is adopted in the QCD design in our work.

2) Characterizing the frequency response of the QCLs and the detectors with electrical rectification

Electrical rectification is an approach to probe the electronic aspect of a nonlinear device, e.g., the inter-subband heterostructures. As the electronic properties of the end product are highly dependent on its geometrical design, it is preferrable to characterize the dynamic behaviour of optoelectronics and ensure they meet the required specifications prior to building complex alignment setups. The electrical rectification method relies on inherent nonlinear characteristics of the optoelectronic devices under test. The usual circuit used to perform rectification to characterize the bandwidth of the QCD and QWIP is depicted in Supplementary Fig. 4a**.** A DC source (Keithley 2450 SourceMeter) is used to apply a bias voltage (V_{DC}) through a 45 GHz bandwidth bias-tee (SHF BT45), which combines with a RF signal ($V_{AC} \cdot cos(\omega_0 t)$) generated from an RF synthesizer (Anritsu MG 3693B). The total voltage generated is then $V_{tot} = V_{DC} + V_{AC} \cdot \cos(\omega_0 t)$. The voltage is applied to the detectors under test with a transmission line of impedance Z_0 = 50 Ω. The reflection

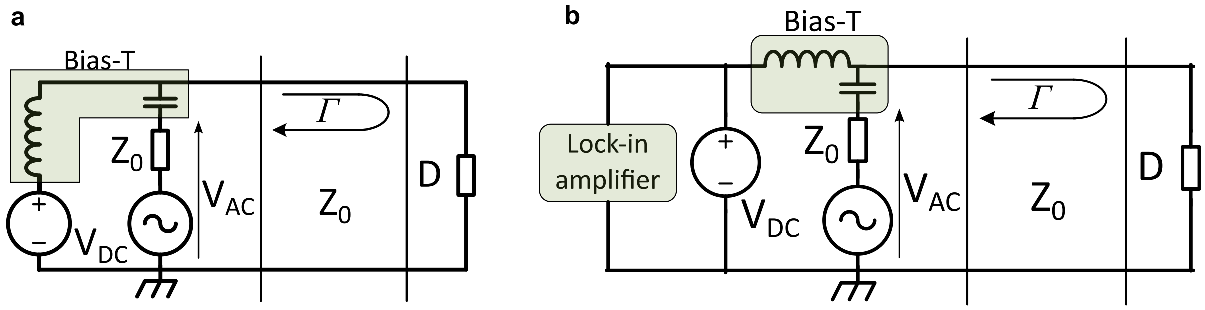

Supplementary Fig. 4. Electrical rectification for bandwidth measurements. a, The electronic circuit used to perform electrical rectification for characterizing the bandwidth of the QCD and the QWIP. DC and AC voltage sources are connected to the dipole D, i.e., the QCD/QWIP under test, through a bias-tee and a transmission line of characteristic impedance Z_0 = 50Ω and reflection coefficient Γ. b, The electronic circuit used to perform electrical rectification for characterizing the QCLs, in which case the DC generator provides a high output current to the device. The AC voltage now carries an extra modulation at very low frequency ω_1. A high-impedance lock-in amplifier is added besides the DC generator to analyse the low frequency signal.

coefficient due to impedance mismatch between the detectors and the transmission line is denoted as $\Gamma(\omega_0)$. When operating at linear I-V characteristic region centred around V_{DC}, the current voltage relation can be simply expressed as $I(V) = I(V_{DC}) + I'(V - V_{DC})$, where $I' = \frac{dI}{dV}$ is the slope of the I-V curve around V_{DC}. In this condition, the complex AC voltage applied onto the detector can be directly related to the transfer function of the device, expressed as:

$$V(\omega_0) - V_{DC} = V_{AC}\big(1 + \Gamma(\omega_0)\big) = V_{AC}\frac{2Z(\omega_0)}{Z_0 + Z(\omega_0)} = V_{AC}H(\omega_0)e^{i\phi(\omega_0)t} \tag{1}$$

thus:

$$H(\omega_0)e^{i\phi(\omega_0)t} = 1 + \Gamma(\omega_0) \tag{2}$$

where $\phi(\omega_0)$ is the phase response. In such a linear condition, it is not trivial to obtain the response of the device without using a coherent linear detector, such as a vector network analyser (VNA), which can directly measure the complex reflection coefficient. Therefore, a way to walk around it is to configure the level of the bias in such a way that the detectors operate in the nonlinear I-V characteristic region, generating a second order term. The I-V characteristic becomes:

$$I(V) = I(V_{DC}) + I'(V - V_{DC}) + I''(V - V_{DC})^2 \tag{3}$$

thus:

$$\begin{aligned} I(V) - I(V_{DC}) &= I'H(\omega_0)V_{AC}\cos\big(\omega_0 t + \phi(\omega_0)\big) \\ &\quad + \frac{1}{2}I''\Big(H(\omega_0)V_{AC}\cos\big(\omega_0 t + \phi(\omega_0)\big)\Big)^2 \\ &= I'H(\omega_0)V_{AC}\cos\big(\omega_0 t + \phi(\omega_0)\big) \\ &\quad + \frac{1}{4}I''H(\omega_0)^2{V_{AC}}^2\Big(1 + \cos\big(2\omega_0 t + 2\phi(\omega_0)\big)\Big) \end{aligned} \tag{4}$$

One can see that the second order term rectifies the applied AC voltage and generates rectified DC current, expressed as:

$$I_d = \frac{1}{4}I''H(\omega_0)^2{V_{AC}}^2 \tag{5}$$

which is proportional to the response of the device $H(\omega_0)^2$. For characterization of the QCD and QWIP in this experiment, the rectified current is normally in the order of a few μA, which can be directly measured by the DC SourceMeter.

In the case of characterizing the bandwidth of the QCL, which is an active component requiring DC current of over hundreds of mA, much higher than the rectified current, hindering accurate measurements. Therefore, the electrical rectification circuit is adapted with an extra low frequency modulation ($\omega_1 \ll \omega_0$), which is to be measured as the rectified current. The adapted circuit is shown in Supplementary Fig. 4b**.** A high input impedance lock-in amplifier is used to analyse the rectified current centred at ω_1, which is proportional to the response of the QCL $H(\omega_0)^2$.

During the measurements, the RF frequency ω_0 is tuned to capture the frequency response across the operational range of all the devices. It is worth noting that such electrical rectification method can only be used to characterize the amplitude response, as the phase response is missing after the square operation. Linear coherent detection will be needed to recover the phase response, which cannot be performed with this simple setup.

3) The details of digital signal processing (DSP) at transmitter and receiver

The transmitter- and receiver-side DSP are performed in MATLAB, and a block diagram describing the DSP routine can be found in Supplementary Fig. 5. At the transmitter, we generate a pseudo-random binary sequence (PRBS) of over 1 million unrepeated bits in length, utilizing the Mersenne Twister with a shuffled seed number for every test case. The bit-sequence is mapped into symbols of different modulation formats, i.e., NRZ, PAM4 and PAM6. The symbols are up-sampled to 4 samples per symbol and pulse-shaped with a root-raise-cosine (RRC) filter with a roll-off factor between 0.1 and 0.3, optimized for each test case. After that the signal is re-sampled to match the sampling rate of the arbitrary waveform generator (AWG), which is 50 GS s^{-1}. Finally, a static 2-tap finite impulse response (FIR) pre-emphasis filter with Z-domain transfer function $H_{pre\text{-}emphasis}(Z) = 1 + \alpha Z^{-1}$, where α is the pre-emphasis coefficient with adjustable values from 0 to -1. $\alpha = 0$ means no pre-emphasis, and $\alpha = -1$ corresponds to a maximum pre-emphasis with a substantial enhancement of the high-frequency response.

After the FSO transmission link, the detected signal is converted back to the digital domain and is processed with receiver DSP. The signal firstly passes through an RRC filter with identical roll-off as the transmitter as a matched filtering, then being upsampled to 4 sample per symbol for

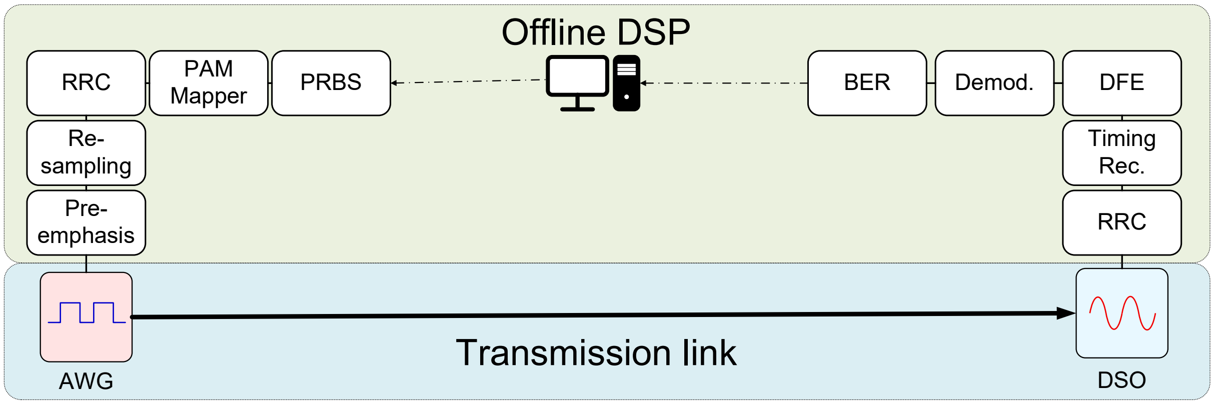

Supplementary Fig. 5. The DSP routine at both transmitter and receiver.

timing recovery, which is based on the maximum variance method. After the timing recovery the signal is down-sampled to 1 sample per symbol and processed with a data-aided symbol-spaced adaptive decision-feedback equalizer (DFE). As this experiment focuses on maximizing the performance of highest achievable symbol rate for various modulation formats, we used 99 feedforward taps and 99 feedback taps for all the test cases during the measurements. The complexity of the DFE can be potentially reduced with fewer taps at the cost of worsened bit-error rate (BER). After the DFE, the recovered symbols are demodulated into bits, and the BER is counted by comparing the recovered bit sequence with the generated PRBS at the transmitter. Each received signal trace consists of more than 1 million symbols for BER calculation.

4) Link budget analysis comparing LWIR with the near-IR telecom band

In the context of LWIR FSO transmission links, there is a keen interest in applications spanning considerable distances, often extending to several kilometres for both terrestrial and space applications. The efficacy of optical systems over these vast lengths can be ascertained using the link budget. This metric calculates the power received at the detector, considering factors such as the initial emitted power, the dimensions of the optics employed, and the attenuation experienced by the beam during its propagation. Here we perform link budget analysis based on the 9-μm wavelength beams. When transmitting through the atmosphere, the FSO beam degrades due to several factors: 1) beam divergence, which can be predicted by Gaussian optics; 2) absorption by atmosphere gas molecules, e.g., carbon dioxide; 3) particles-induced scattering; and 4) wavefront deformation due to turbulence.

The Gaussian beam radius *ω(z)* when propagating through free space over distance of *z* can be calculated as:

$$\omega(z) = \omega_0 \sqrt{1 + \left(\frac{\lambda z}{\pi \omega_0^2}\right)} \tag{6}$$

where ω_0 is the beam waist at the source and λ is the wavelength. In our experiment, the initial beam waist radius is approximately 17.2 mm (half inch) after the collimator. When comparing with the 1.55 μm telecom wavelength, the 9 μm beam experienced faster divergence as shown in Supplementary Fig. 6. Considering the initial beam radius of 12.7 mm, equivalent to half an inch, as used in this experiment, the beam radius widens to 25.9 mm after a 100-metre propagation and further to 225.9 mm after 1 kilometre, posing challenges in capturing all the beam energy. Assuming a larger transmitter aperture, this beam divergence becomes less noticeable. Supplementary Fig. 6 (b) and (c) illustrate the beam radius divergence with initial radii of 25.4 mm (1 inch) and 50.8 mm (2 inches), respectively, showing clearly reduced beam radii after

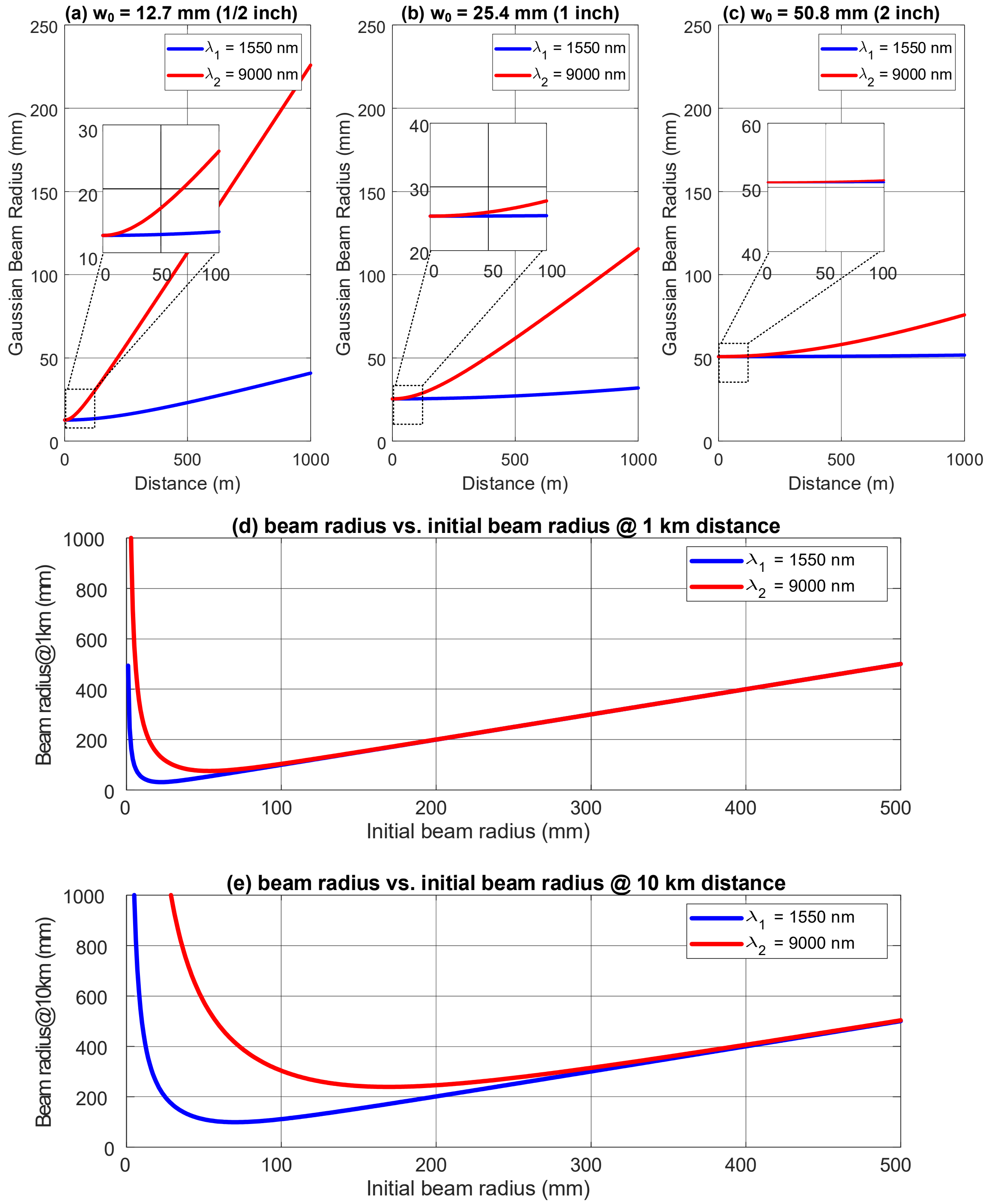

Supplementary Fig. 6. Gaussian beam divergence when propagating in free space. (a)-(c), beam radii divergence as a function of propagation distance with initial beam radius of ½ inch, 1 inch and 2 inches, respectively. Beam radii after propagation vs. initial beam radii after 1 km and 10 km distance are shown in (d) and (e), respectively.

---

propagation. To provide a broader overview, Supplementary Fig. 6 (d) and (e) display the beam

radius as a function of the initial beam radius after propagating distances of 1 km and 10 km, respectively. It is evident that for 1-km FSO transmission, the difference in beam radii between 1.55 μm and 9 μm beams narrows with an initial beam radius of 100 mm. For 10-km transmissions, the point of convergence increases up to approximately 300 mm. Therefore, a critical consideration for practical LWIR FSO is the transceiver's aperture size. Depending on the application scenario, it's important to select an appropriate initial beam size to prevent excessive beam divergence over the relevant distance. It is also noted that a promising approach could involve the use of transmitter arrays, like MIMO antenna arrays in RF wireless communications. These arrays can synthetically create a larger effective aperture, potentially reducing the divergence below that of a single emitter at the same wavelength.

As the turbulence-induced beam profile degradation and wandering are more dynamic and complex, for which dedicated studies are required, here we narrow down our focus to a Gaussian beam propagating through the atmosphere only the first three factors mentioned above. The received optical power can be written as[10]:

$$P_r = P_{Tx} \frac{D_{Rx}^2}{(D_{Tx}+\theta_{div}L)^2} e^{-\gamma(\lambda)L} \tag{7}$$

where P_{Tx} and P_r are transmitted power from the source and received power at the detector, and D_{Tx} and D_{Rx} are the diameters of the transmitter and the receiver, respectively. λ is the wavelength, L is the propagation distance and $\theta_{div} = \frac{\lambda}{\pi\omega_0}$ is the beam divergence with ω_0 the beam waist at the origin. $\gamma(\lambda)$ represents the attenuation caused by the atmosphere. $\gamma(\lambda)$ can be decomposed to be $\gamma(\lambda)=\alpha_m(\lambda)+ \alpha_a(\lambda)+\beta_m(\lambda)+ \beta_a(\lambda)$, where α_m and α_a are the absorption losses of molecules and aerosols, and β_m and β_a are their respective diffusion losses. At a 9 μm wavelength, as utilized in this free-space transmission study, gas absorption is negligible. This leaves Mie and geometric scatterings as the major sources of attenuation. Geometric scattering occurs due to the presence of

large particles such as snow, rain, or dust along the signal path. Although there exist empirical models for such scatterings[11], their average size exceeds the wavelengths of both near-IR and LWIR, their contribution to attenuation is quite similar across these wavelengths. Therefore, the main difference between the NIR and the LWIR FSO when propagating through the atmosphere narrows down to the Mie scattering, caused by the elastic scattering of light in the direction of propagation by particles with dimensions similar to the radiation wavelength (such as fog or haze). Per the empirical models[12,13], it can be described as:

$$\beta_a(\lambda) = \frac{3.91}{V}\left(\frac{\lambda}{\lambda_0}\right)^{-q} \qquad \text{for NIR} \qquad (8)$$

$$\beta_a(\lambda) = \mathrm{A}\mathrm{V}^{b} \qquad \text{for MIR} \qquad (9)$$

where λ_0 is the visibility range reference wavelength (550 nm), V is the visibility range in km, q, A, and b are phenomenological coefficients determined by fitting experimental measurements. Supplementary Table 1 shows the beam attenuation induced by Mie scattering for 1.55 µm and 9 µm for different visibility conditions, based on a similar table presented in the reference[10]. One can see that the 9 µm wavelength window outperforms the 1.55 µm telecom band in all categories by at least a factor of three.

Supplementary Table 1. Beam attenuation caused by Mie scattering

	Attenuation ($dB\cdot km^{-1}$)	
Visibility (km)	1.55 µm	9 µm
20 (Clear)	2.2×10^{-1}	2.2×10^{-2}
5 (Haze)	1.21	0.21
2 (Mist)	4.0	1.1
1 (Fog)	9.3	3.4

In summary, while the 9 μm beam demands larger transmitter and receiver apertures to remain collimated during propagation compared to the 1.55 μm beam, it experiences significantly less attenuation caused by Mie scattering under various visibility conditions.

5) FSO system characterisation results

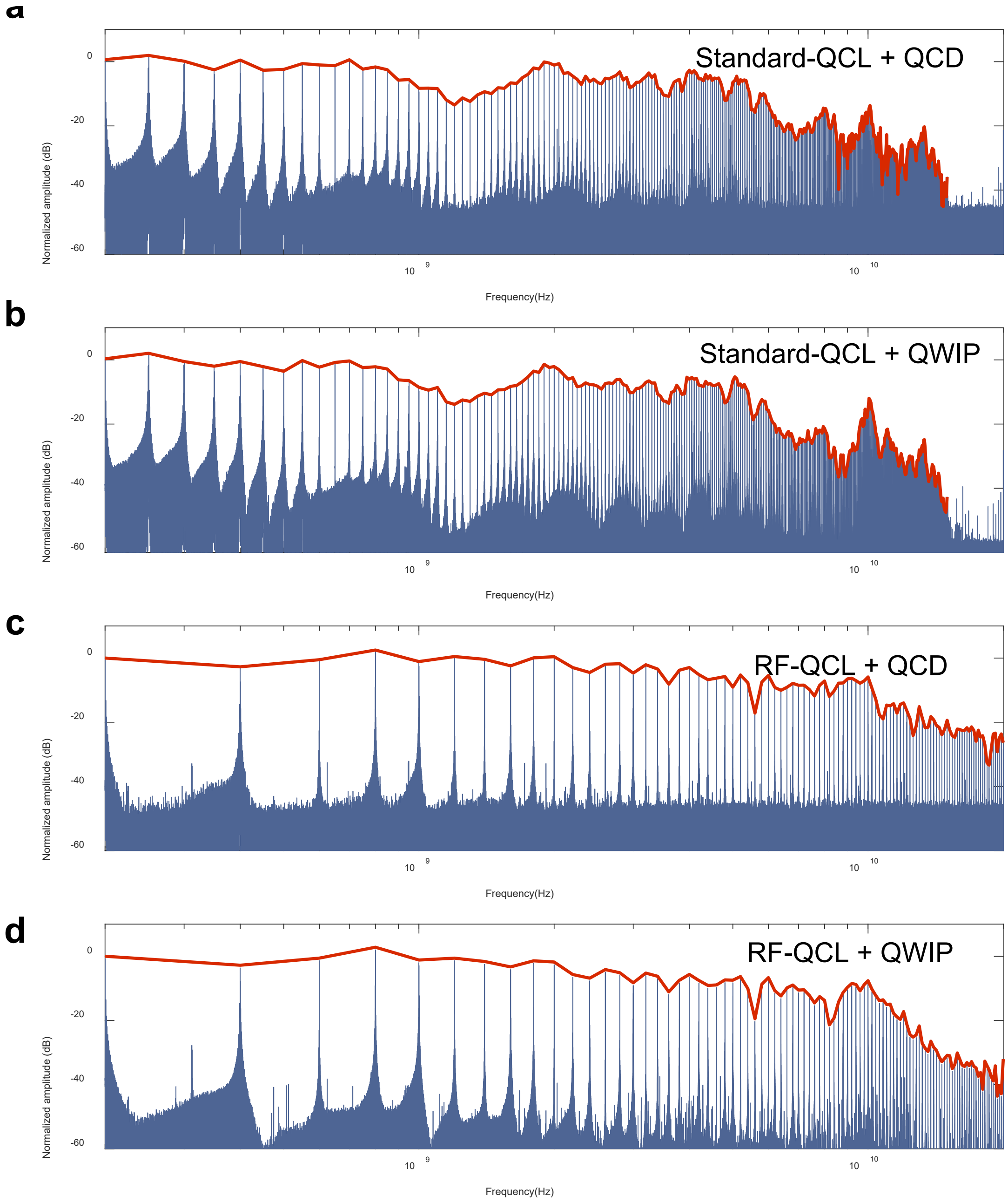

Supplementary Fig. 7. System end-to-end amplitude frequency response calibration with frequency combs. a, Frequency response of Standard-QCL with QCD calibrated up to 15 GHz. b, Frequency response of Standard-QCL with QWIP calibrated up to 15 GHz. c, Frequency response of RF-QCL with QCD calibrated up to 20 GHz. d, Frequency response of RF-QCL with QWIP calibrated up to 20 GHz.

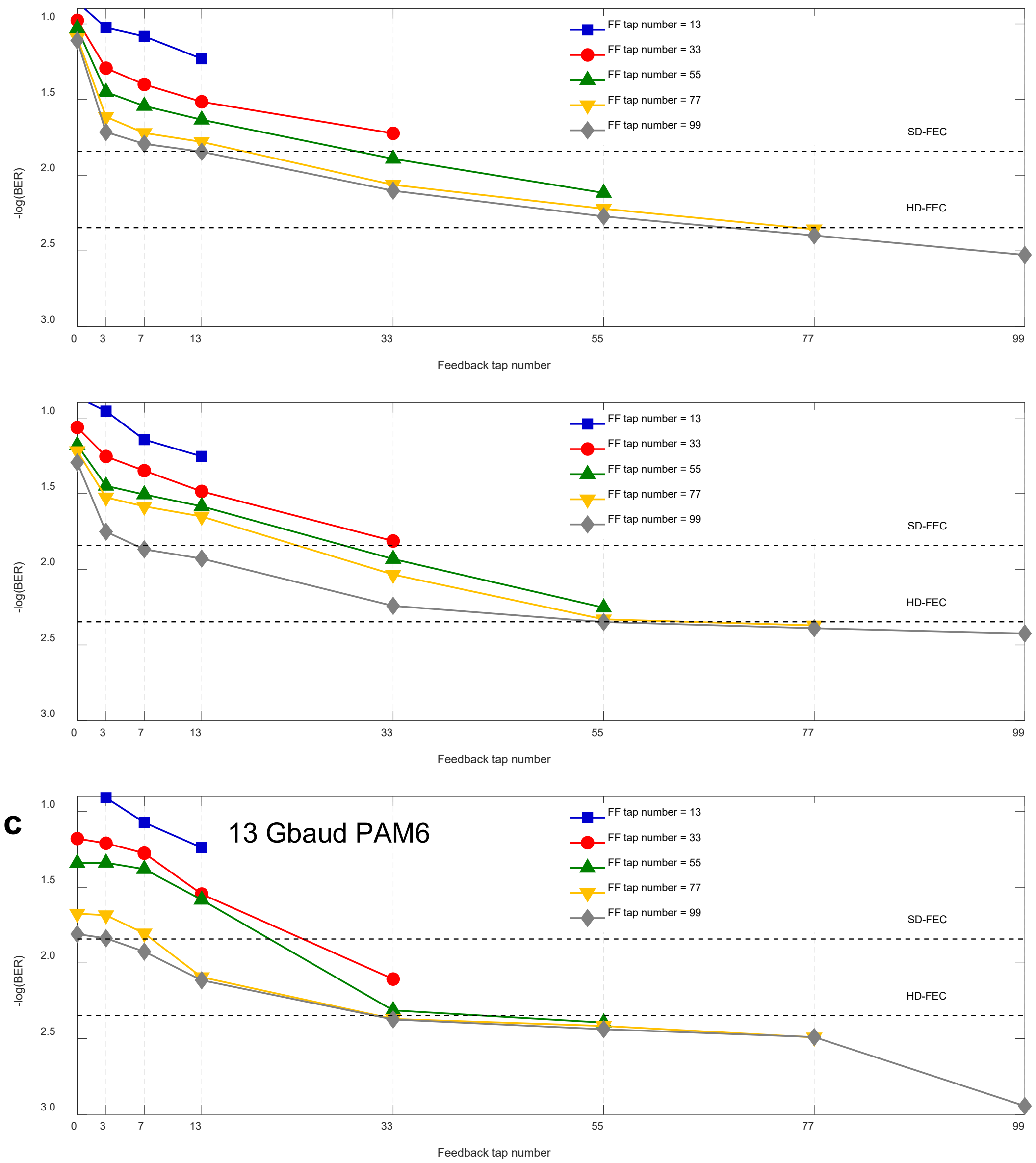

Supplementary Fig. 8. BER performance as a function of DFE tap number when using the Standard-QCL and the QCD. a, 33 Gbaud NRZ. In this case, the 6.25% overhead HD-FEC limit can be achieved with DFE of 77 feedforward (FF) taps plus 77 feedback (FB) taps. b, 18 Gbaud PAM4. In this case, the HD-FEC limit can be achieved with DFE of 77 FF taps plus 77 FB taps. c, 13 Gbaud PAM6. The HD-FEC can be achieved with either 55 FF taps plus 55 FB taps, or 77 FF taps plus 33 FB taps.

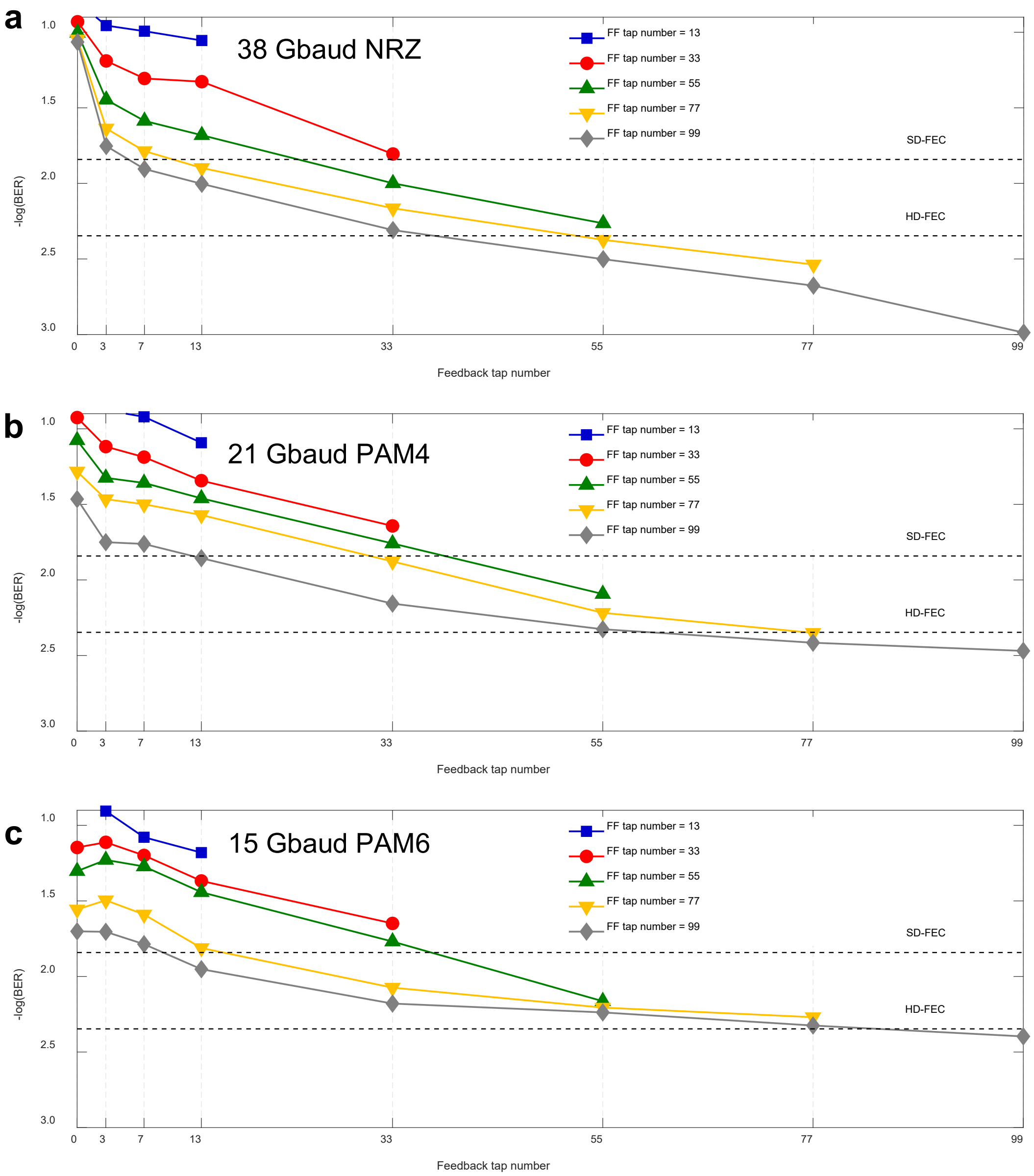

Supplementary Fig. 9. BER performance as a function of DFE tap number when using the Standard-QCL and the QWIP. a, 38 Gbaud NRZ. In this case, the 6.25% overhead HD-FEC limit can be achieved with DFE of 77 FF taps plus 55 FB taps. b, 21 Gbaud PAM4. In this case, the HD-FEC limit can be achieved with DFE of 99 FF taps plus 77 FB taps. c, 15 Gbaud PAM6. The HD-FEC can only be achieved with 99 FF taps plus 99 FB taps.

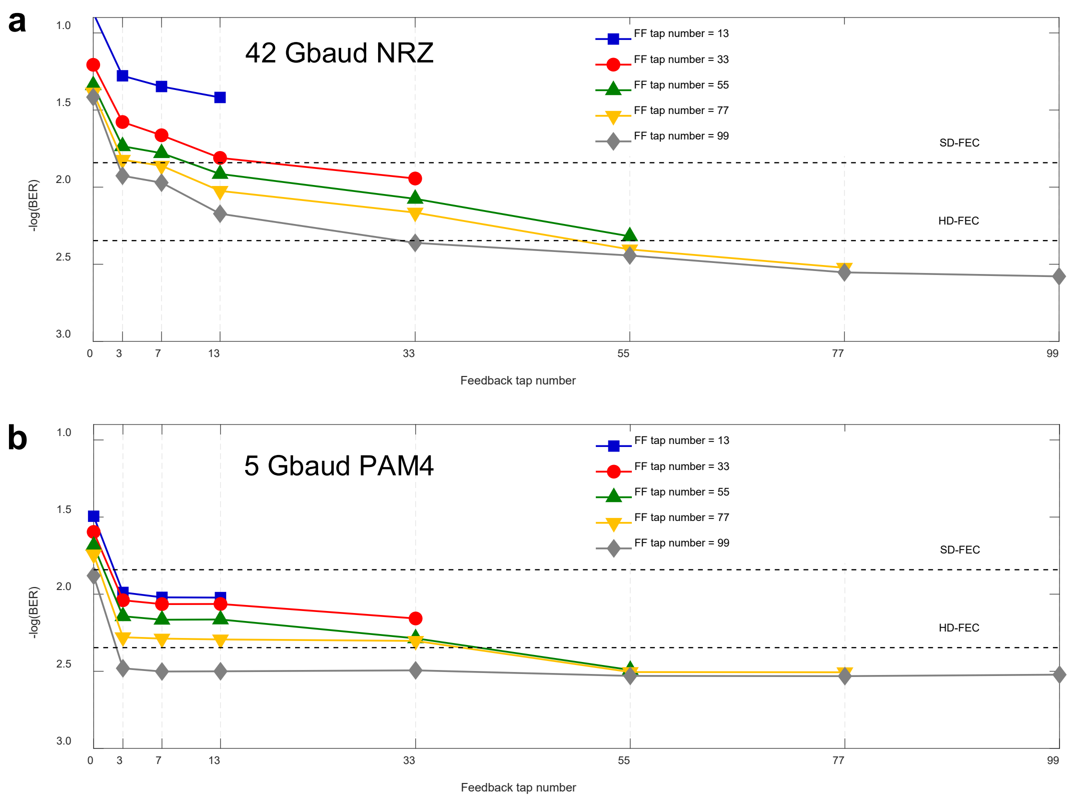

Supplementary Fig. 10. BER performance as a function of DFE tap number when using the RF-QCL and the QCD. a, 42 Gbaud NRZ. In this case, the 6.25% overhead HD-FEC limit can be achieved with DFE of 77 FF taps plus 55 FB taps. b, 5 Gbaud PAM4. In this case, the HD-FEC limit can be achieved with DFE of either 99 FF taps plus 3 FB taps, or 55 FF plus 55 FB taps.

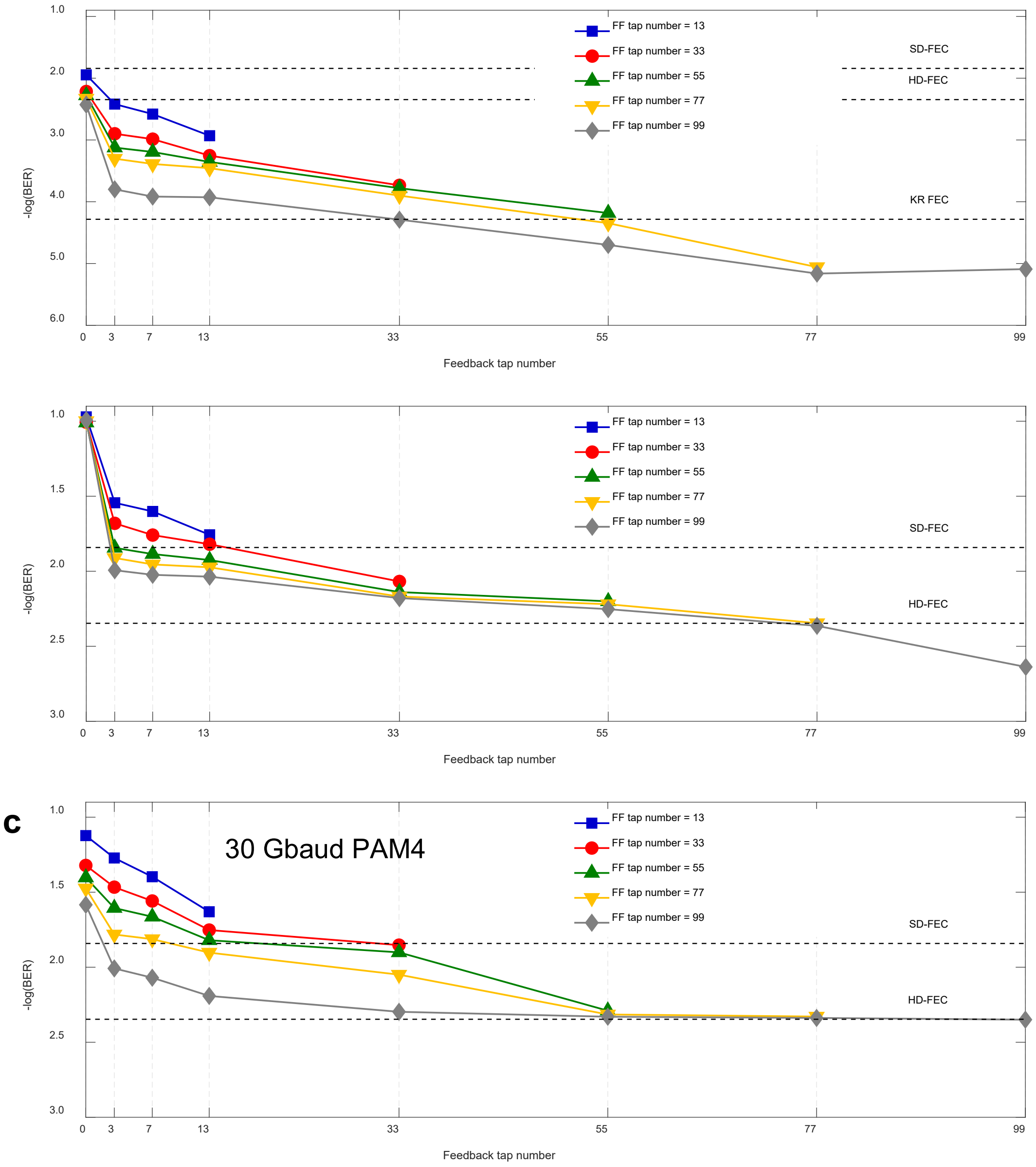

Supplementary Fig. 11. BER performance as a function of DFE tap number when using the RF-QCL and the QWIP. a, 40 Gbaud NRZ. In this case, the 6.25% overhead HD-FEC limit can be achieved with DFE of 13 FF taps plus 3 FB taps, and the KR-FEC limit can be achieved with DFE of either 77 FF taps plus 55 FB taps, or 99 FF taps plus 33 FB taps. b, 55 Gbaud NRZ. In this case, the HD-FEC limit can be achieved with DFE of 77 FF taps plus 77 FB taps. c, 30 Gbaud PAM4. In this case, the minimum required number of taps for DFE are 99 FF taps plus 99 FB taps to achieve the HD-FEC limit.

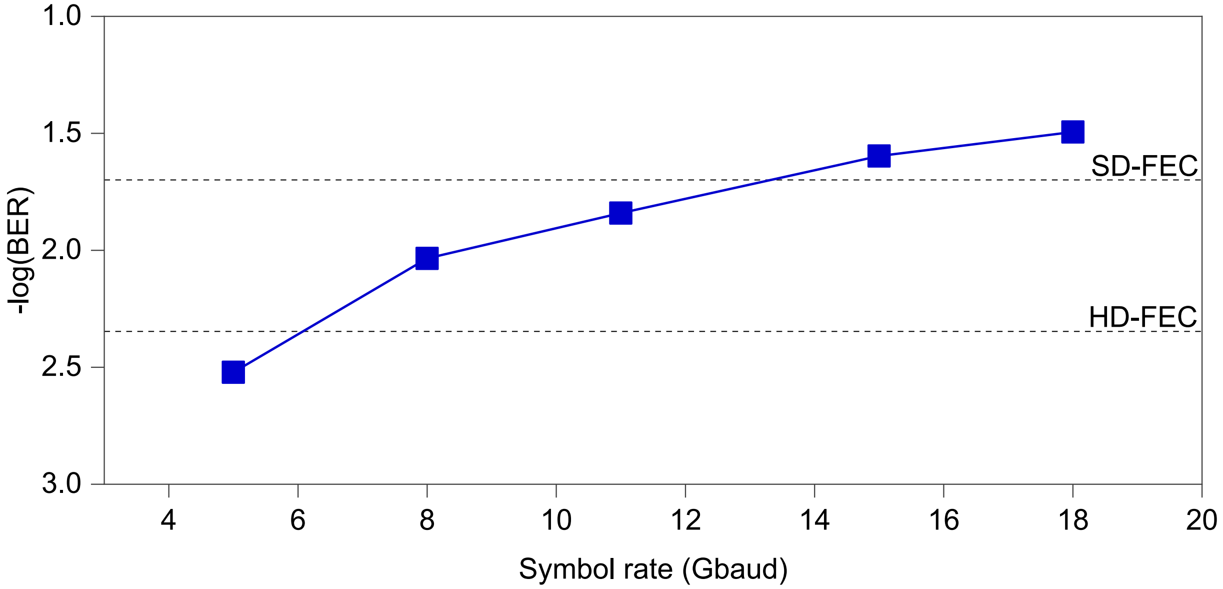

Supplementary Fig. 12. BER performance as a function of symbol rate for PAM4 signals using the RF-QCL and the QCD. All BER values are measured at bias current of 480 mA. It can be noted that as the symbol rate increases, the increase in the BER is relatively gradual, indicating the system is more noise-limited than bandwidth-limited. 11 Gbaud PAM4 can be achieved below the SD-FEC limit.

6) State of the art table in free-space optical communications with directly modulated quantum cascade lasers and interband cascade lasers.

Here, we provide a concise summary of experimental demonstrations involving directly modulated QCL- and interband cascade laser (ICL)-based free-space transmission, as listed in Supplementary Table 2. We list critical specifications including wavelength, transmitted signal format and speed, the operational temperature of the laser source, and the detector configuration. Note that while this summary is not exhaustive, it aims to represent the current state of the art and the position of our work in the research of directly modulated QCL/ICL-based free-space optical communications in a clear manner.

Supplementary Table 2. Experimental demonstrations of directly modulated QCL- and ICL-based free-space transmissions with >1 THz carrier frequencies

Wavelength	Signal [a]	QCL/ICL temperature	Detector	Ref
7.3 μm	10 MHz analogue [b]	80 K	Liquid nitrogen cooled MCT	Ref 14
8.1 μm	2.5 Gb/s NRZ	85 K	QWIP at 77K	Ref 15
8.1 μm	1.5 GHz QPSK	25 K	Liquid nitrogen cooled MCT	Ref 16
9.3 μm	330 MHz / 115 kb/s analogue / NRZ [c]	288 K	Room-temperature MCT	Ref 17
10.46 μm	20 kHz PFM	240 K-340 K	Liquid nitrogen cooled MCT	Ref 18
3 μm	70 Mb/s NRZ	77 K ICL	Thermoelectric cooled MCT	Ref 19
73 μm	580 kHz analogue	10 K	THz QWP at 4 K	Ref 20
72.6 μm	1 Mb/s NRZ	13 K	THz QWP at 4 K	Ref 21
77 μm	5 Mb/s NRZ	10 K	THz QWP at 4 K	Ref 22
92 μm	20 Mb/s NRZ	11 K	THz QWP at 3.5 K	Ref 23
4.7 μm	40 MHz analogue video	Room-temperature [c]	Room-temperature MCT	Ref 24
Mid-IR [d]	20 MHz PPM	Uncooled [c]	Uncooled MCT	Ref 25
10.6 μm	1 Gb/s NRZ	298 K	MCT at 230 K	Ref 26
4 μm	680 Mb/s RZ	288 K	Room-temperature MCT	Ref 27
4.65 μm	3 Gb/s NRZ, PAM4/8	293 K	MCT at 200 K	Ref 28
4.65 μm	4 Gb/s PAM4, DMT	293 K	MCT at 200 K	Ref 29
4.65 μm	6 Gb/s PAM8	293 K	MCT at 200 K	Ref 30
9.15 μm	8.1 Gb/s PAM8	288 K	MCT at 200 K	Ref 31
9.6 μm	11 Gb/s PAM4	273 K	Uncooled QCD	Ref 32
4.18 μm	14 Gb/s PAM4	293 K ICL	Uncooled ICIP	Ref 33
9.15 μm	16.9 Gb/s PAM4	288K	MCT at 200 K	Ref 34
9.15 μm	36 Gb/s PAM4	288 K	Uncooled QCD	This work
9.15 μm	42 Gb/s PAM4	288 K	Uncooled QWIP	This work
9.14 μm	42 Gb/s NRZ	288K	Uncooled QCD	This work
9.14 μm	60 Gb/s PAM4	288K	Uncooled QWIP	This work

ICL, interband cascade laser; MCT, mercury-cadmium-telluride detector; QWP: quantum-well photodetector; ICIP, interband cascade infrared photodetector; NRZ, non-return-to-zero; QPSK, quadrature phase shift keying; PFM, pulsed frequency modulation; PPM, pulse positioning modulation; PAM4 / PAM8, 4- / 8-level pulse amplitude modulation; DMT, discrete multitone

[a] Digital signals' speed benchmarked against HD-FEC limit
[b] modulated on to a 66 MHz carrier
[c] operation temperature not explicitly specified
[d] wavelength not explicitly specified.

REFERENCES

1. Faist J, Capasso F, Sivco DL, Sirtori C, Hutchinson AL, Cho AY. Quantum Cascade Laser. *Science* 1994, **264**(5158)**:** 553-556.
2. Esaki L, Tsu R. Superlattice and Negative Differential Conductivity in Semiconductors. *IBM J Res Dev* 1970, **14**(1)**:** 61-65.
3. Kazarinov RF, Suris RA. Possibility of amplication of electromagnetic waves in a semiconductor with a superlattice. *Sov Phys Semicond* 1971, **5**(4)**:** 707-709.
4. Levine BF, Choi KK, Bethea CG, Walker J, Malik RJ. New 10 μm infrared detector using intersubband absorption in resonant tunneling GaAlAs superlattices. *Applied Physics Letters* 1987, **50**(16)**:** 1092-1094.
5. Palaferri D, Todorov Y, Bigioli A, Mottaghizadeh A, Gacemi D, Calabrese A*, et al.* Room-temperature nine-μm-wavelength photodetectors and GHz-frequency heterodyne receivers. *Natur* 2018, **556**(7699)**:** 85-88.
6. Hofstetter D, Beck M, Faist J. Quantum-cascade-laser structures as photodetectors. *Applied Physics Letters* 2002, **81**(15)**:** 2683-2685.
7. Gendron L, Carras M, Huynh A, Ortiz V, Koeniguer C, Berger V. Quantum cascade photodetector. *Applied Physics Letters* 2004, **85**(14)**:** 2824-2826.
8. Reininger P, Schwarz B, Detz H, MacFarland D, Zederbauer T, Andrews AM*, et al.* Diagonal-transition quantum cascade detector. *Applied Physics Letters* 2014, **105**(9).
9. Quinchard G, Mismer C, Hakl M, Pereira J, Lin Q, Lepillet S*, et al.* High speed, antenna-enhanced 10.3 μm quantum cascade detector. *Applied Physics Letters* 2022, **120**(9).
10. Trichili A, Cox MA, Ooi BS, Alouini M-S. Roadmap to free space optics. *J Opt Soc Am B* 2020, **37**(11)**:** A184-A201.
11. Esmail MA, Fathallah H, Alouini M-S. An Experimental Study of FSO Link Performance in Desert Environment. *IEEE Communications Letters* 2016, **20**(9)**:** 1888-1891.
12. Korevaar EJ, Kim II, McArthur B, Korevaar EJ. Comparison of laser beam propagation at 785 nm and 1550 nm in fog and haze for optical wireless communications. *Optical Wireless Communications III*; 2001. pp. 26-37.
13. Nebuloni R. Empirical relationships between extinction coefficient and visibility in fog. *Applied Optics* 2005, **44**(18)**:** 3795-3804.
14. Martini R, Gmachl C, Falciglia J, Curti FG, Bethea CG, Capasso F*, et al.* High-speed modulation and free-space optical audio/video transmission using quantum cascade lasers. *Electronics Letters* 2001, **37**(3)**:** 191-193.
15. Capasso F, Paiella R, Martini R, Colombelli R, Gmachl C, Myers TL*, et al.* Quantum cascade lasers: ultrahigh-speed operation, optical wireless communication, narrow linewidth, and far-infrared emission. *IEEE Journal of Quantum Electronics* 2002, **38**(6)**:** 511-532.
16. Martini R, Bethea C, Capasso F, Gmachl C, Paiella R, Whittaker EA*, et al.* Free-space optical transmission of multimedia satellite data streams using mid-infrared quantum cascade lasers. *Electronics Letters* 2002, **38**(4)**:** 181-183.
17. Blaser S, Hofstetter D, Beck M, Faist J. Free-space optical data link using Peltier-cooled quantum cascade laser. *Electronics Letters* 2001, **37**(12)**:** 778-780.
18. Taslakov M, Mecherle S, Simeonov V, van den Bergh H. Line-of-sight data transmission system based on mid IR quantum cascade laser. Free-Space Laser Communication Technologies XX; 2008; 2008.
19. Soibel A, Wright MW, Farr WH, Keo SA, Hill CJ, Yang RQ*, et al.* Midinfrared Interband Cascade Laser for Free Space Optical Communication. *IPTL* 2010, **22**(2)**:** 121-123.
20. Chen Z, Tan ZY, Han YJ, Zhang R, Guo XG, Li H*, et al.* Wireless communication demonstration at 4.1 THz using quantum cascade laser and quantum well photodetector. *Electronics Letters* 2011, **47**(17).
21. Zhiyong Tan ZT, Zhen Chen ZC, Juncheng Cao JC, Huichun Liu HL. Wireless terahertz light transmission based on digitally-modulated terahertz quantum-cascade laser. *Chinese Optics Letters* 2013, **11**(3)**:** 031403-031405.

22. Chen Z, Gu L, Tan Z, Wang C, Cao J. Real-time video signal transmission over a terahertz communication link. *Chinese Optics Letters* 2013, **11**(11)**:** 112001-112003.
23. Gu L, Tan Z, Wu Q, Wang C, Cao J. 20 Mbps wireless communication demonstration using terahertz quantum devices. *Chinese Optics Letters* 2015, **13**(8)**:** 081402-081404.
24. Liu C, Zhai S, Zhang J, Zhou Y, Jia Z, Liu F*, et al.* Free-space communication based on quantum cascade laser. *Journal of Semiconductors* 2015, **36**(9).
25. Turner MD, Kamerman GW, Wasiczko Thomas LM, Spillar EJ, Luzhansky E, Choa F-S*, et al.* Mid-IR free-space optical communication with quantum cascade lasers. *Laser Radar Technology and Applications XX; and Atmospheric Propagation XII*; 2015.
26. Liu JJ, Stann BL, Klett KK, Cho PS, Pellegrino PM, van Eijk AM*, et al.* Mid and long-wave infrared free-space optical communication. Laser Communication and Propagation through the Atmosphere and Oceans VIII; 2019; 2019. p. 1113302.
27. Spitz O, Didier P, Durupt L, Diaz-Thomas DA, Baranov AN, Cerutti L*, et al.* Free-Space Communication With Directly Modulated Mid-Infrared Quantum Cascade Devices. *IEEE Journal of Selected Topics in Quantum Electronics* 2022, **28**(1)**:** 1-9.
28. Pang X, Ozolins O, Schatz R, Storck J, Udalcovs A, Navarro JR*, et al.* Gigabit free-space multi-level signal transmission with a mid-infrared quantum cascade laser operating at room temperature. *Opt Lett* 2017, **42**(18)**:** 3646-3649.
29. Pang X, Ozolins O, Zhang L, Schatz R, Udalcovs A, Yu X*, et al.* Free-Space Communications Enabled by Quantum Cascade Lasers. *physica status solidi (a)* 2021, **218**(3)**:** 2000407.
30. Pang X, Schatz R, Joharifar M, Udalcovs A, Bobrovs V, Zhang L*, et al.* Direct Modulation and Free-Space Transmissions of up to 6 Gbps Multilevel Signals With a 4.65-µm Quantum Cascade Laser at Room Temperature. *J Lightwave Technol* 2022, **40**(8)**:** 2370-2377.
31. Han M, Joharifar M, Wang M, Schatz R, Puerta R, Sun Y-T*, et al.* High Spectral Efficiency Long-Wave Infrared Free-Space Optical Transmission With Multilevel Signals. *J Lightwave Technol* 2023, **41**(20)**:** 6514-6520.
32. Joharifar M, Dely H, Pang X, Schatz R, Gacemi D, Salgals T*, et al.* High-Speed 9.6-µm Long-Wave Infrared Free-Space Transmission With a Directly-Modulated QCL and a Fully-Passive QCD. *J Lightwave Technol* 2023, **41**(4)**:** 1087-1094.
33. Didier P, Knötig H, Spitz O, Cerutti L, Lardschneider A, Awwad E*, et al.* Interband cascade technology for energy-efficient mid-infrared free-space communication. *Photonics Research* 2023, **11**(4).
34. Joharifar M, Dely H, Durupt L, Ostrovskis A, Schatz R, Puerta R*, et al.* 16.9 Gb/s Single-Channel LWIR FSO Data Transmission with Directly Modulated QCL and MCT Detector. Optical Fiber Communication Conference (OFC) 2024; San Diego, California: p. Th2A.25.
